# The short-term effects of blood donation on the ocular parameters including blood flow of the retina and choroid in healthy people using OCT- angiography

**DOI:** 10.1186/s12886-023-03002-3

**Published:** 2023-06-12

**Authors:** Mengmeng Yu, Xiaolei Sun, Fanxing Zeng, Xiang Gao, Zhenzhen Li, Gongqiang Yuan, Ting Wang

**Affiliations:** 1 Department of Ophthalmology, Eye Hospital of Shandong First Medical University, Shandong First Medical University & Shandong Academy of Medical Sciences, Jinan, China; 2 State Key Laboratory Cultivation Base, Shandong Provincial Key Laboratory of Ophthalmology, Shandong Eye Institute, Jinan, China; 3 School of Ophthalmology, Shandong First Medical University, Jinan, China

**Keywords:** Blood donation, Intraocular pressure, Retina, Choroid, Optical coherence tomography angiography

## Abstract

**Background:**

To investigate the short-term effects of blood donation on the morphology and blood flow of the retina and choroid in healthy people using optical coherence tomography angiography (OCTA).

**Methods:**

Twenty-eight healthy blood donors (56 eyes) who participated in the 200 ml voluntary blood donation between March 2, 2021 and January 20, 2022 were included. The best corrected visual acuity (BCVA), systolic (SBP) and diastolic blood pressure (DBP), intraocular pressure (IOP), subfoveal choroid thickness (SFCT), retinal thickness (RT), retinal superficial vascular density (SVD), deep vascular density (DVD) and foveal avascular were a (FAZ) were measured and statistically analysed 10 min before, 30 min and 24 h after the blood donation.

**Results:**

The 200 ml blood donation could cause significant IOP reduction at 24 h (*P* = 0.006), which was negatively correlated with SBP (*r* = -0.268, P = 0.046), while SBP, DBP, or ocular perfusion pressure were not affected (> 0.05). Moreover, no significant difference existed in the OCT and OCTA indexes, including SFCT, RT, SVD, DVD, and FAZ, before and after the 200 ml blood donation (*P* > 0.05). The visual acuity was not affected either (*P* > 0.05).

**Conclusions:**

The 200 ml blood donation was noted to be associated with statistically significant IOP reduction at 24 h, while SBP, DBP, or OPP was not affected. The blood flow of the retina and choroid or the visual acuity did not change significantly after the blood donation. Larger studies with different volumes of blood donation were needed to further analysis the effect of blood donation on ocular parameters.

## Background

The practice of voluntary blood donation can not only guarantee the needs of clinical blood use, ensure the safety of blood transfusion, and achieve the purpose of treating diseases and saving lives, but it is also a social mutual aid behaviour, which was an important embodiment of the humanitarian spirit. Several clinical studies suggested that blood donation would not jeopardize the donator’s health, on the contrary, it could stimulate the hematopoietic function of the bone marrow and had a positive effect on reducing the risks of cardiovascular and cerebrovascular diseases.

While blood donation causes acute blood loss, the reduction in circulating blood volume leads to a decrease in mean arterial pressure (MAP), followed by a decrease in cardiac output and pulse pressure[1]. With pressure receptor-mediated activation of the sympathetic nervous system, heart rate and peripheral vascular resistance increase to restore MAP, while the ocular perfusion pressure (OPP) in the eye would decrease or not have not been well investigated[2]. Previous studies indicated that the autoregulation mechanism could maintain the relatively constant level of blood flow in the major vascular structure of the retina and choroid[3, 4], while the microcirculation in the macular and subfoveal choroid was still unclear. Additionally, there was growing evidence that blood insufficiency and vascular disorders were the basis for a variety of common eye diseases, such as central serous chorioretinopathy (CSC)[5–7].

Optical coherence tomography angiography (OCTA) is a non-invasive, fast and high-resolution ophthalmic imaging technology that used to detect anatomical abnormalities associated with retinopathies in ophthalmic clinics, and has the ability to assess dynamic retinal vascular changes[8]. Therefore, our study investigated the short-term effects of blood donation on morphology and blood flow of the retina and choroid in healthy people by OCTA and evaluated the changes in intraocular pressure (IOP), subfoveal choroid thickness (SFCT), retinal thickness (RT) and retinal and macular blood flow related quantitative indexes, to get a more comprehensive understanding of the effects of blood donation and blood volume reduction on ocular circulation, and provide a reference both for ophthalmologists and the blood donators.

## Methods

This pilot study complied with the Declaration of Helsinki of 1964 and the Institutional Review Board of the Eye Hospital of Shandong First Medical University had approved the research protocol (SDSYKYY202103). All healthy volunteers who participated in the voluntary blood donation of 200ml at Shandong Eye Hospital on March 2, 2021 and January 20, 2022 were enrolled in this study. All volunteers, with no systemic diseases and no ocular diseases other than refractive error successfully, completed the voluntary blood donation activities between 8 am to 10 am. All included participants enrolled in this study had provided their written informed consent on the enrolment.

Inclusion criteria: [1] age ≥ 18 years; [2] The Snellen best corrected visual acuity (BCVA) ≥ 20/20; [3] IOP ≤ 21 mmHg before blood donation; Exclusion criteria: [1] previous ophthalmic disease or history of ophthalmic surgery; [2] donator with high myopia ≥ 6.0 diopter; [3] any disease that may affect the microvasculature of the eye, such as glaucoma, vitreoretinal diseases, and neurological ocular diseases, et al.; [4] systemic diseases like hypertension, hypotension, diabetes mellitus, or others might affect the microcirculation of the eye; [5] intake of coffee or alcohol within 24 h before the blood donation. Both eyes were included if the volunteers meet the inclusion and exclusion criteria.

The refractive error, BCVA, axial length (AL), systolic blood pressure (SBP), diastolic blood pressure (DBP), IOP, OPP (which refers to the arterial pressure in the ocular vessels minus the IOP, usually calculated as two-thirds of the MAP minus the IOP, that was, OPP = 2/3* MAP-IOP, where MAP = DBP + 1/3*(SBP-DBP))[9], fundus photography, optical coherence tomography (OCT) and optical coherence tomography angiography (OCTA) in the macular were performed 10 min before, 30 min and 24 h after blood donation. We selected 30 min and 24 h after the blood donation as evaluation points, because 30 min could reflect the morphological and blood flow changes of choroid and retina caused by immediate blood loss, and 24 h could reflect the recovery of ocular circulation after self-regulatory mechanism[1].

Visual acuity was examined using the internationally accepted Snellen visual chart and was then converted to the respective LogMAR equivalent for statistical analysis; AL was measured using an IOL-Master (IOL-Master, Carl Zeiss Meditec, Oberkochen, Germany); IOP was measured using a handheld rebound tonometer (SW-500, suoer, Tianjin, China). BP were measured using a semi-automated oscillometric BP recorder (HEM-7012, OMRON Corporation, Japan). For blood pressure (BP) measurement, volunteers were asked to rest for at least 10 min to ensure a stable blood status. Mean arterial pressure (MAP) was calculated as the DBP plus one-third of the difference between the diastolic and SBP. OPP was calculated as two-thirds of the MAP minus the IOP[9]. All of the measurements were taken three times, and the mean value of the three measurements was used for the statistical analysis. OCT and OCTA examinations were performed with an optical coherence tomography scanner, Optovue RX (Optovue Inc. Fremont, Califonia, USA) and SD-OCT (ToPi-Sigma 1000, TUPI, Beijing, China) after pupillary dilation. Volunteers who exhibited clear OCT images were included in the analysis. The above machines were operated and the images were captured by the same doctor (XLS).

**Fig. 1:**
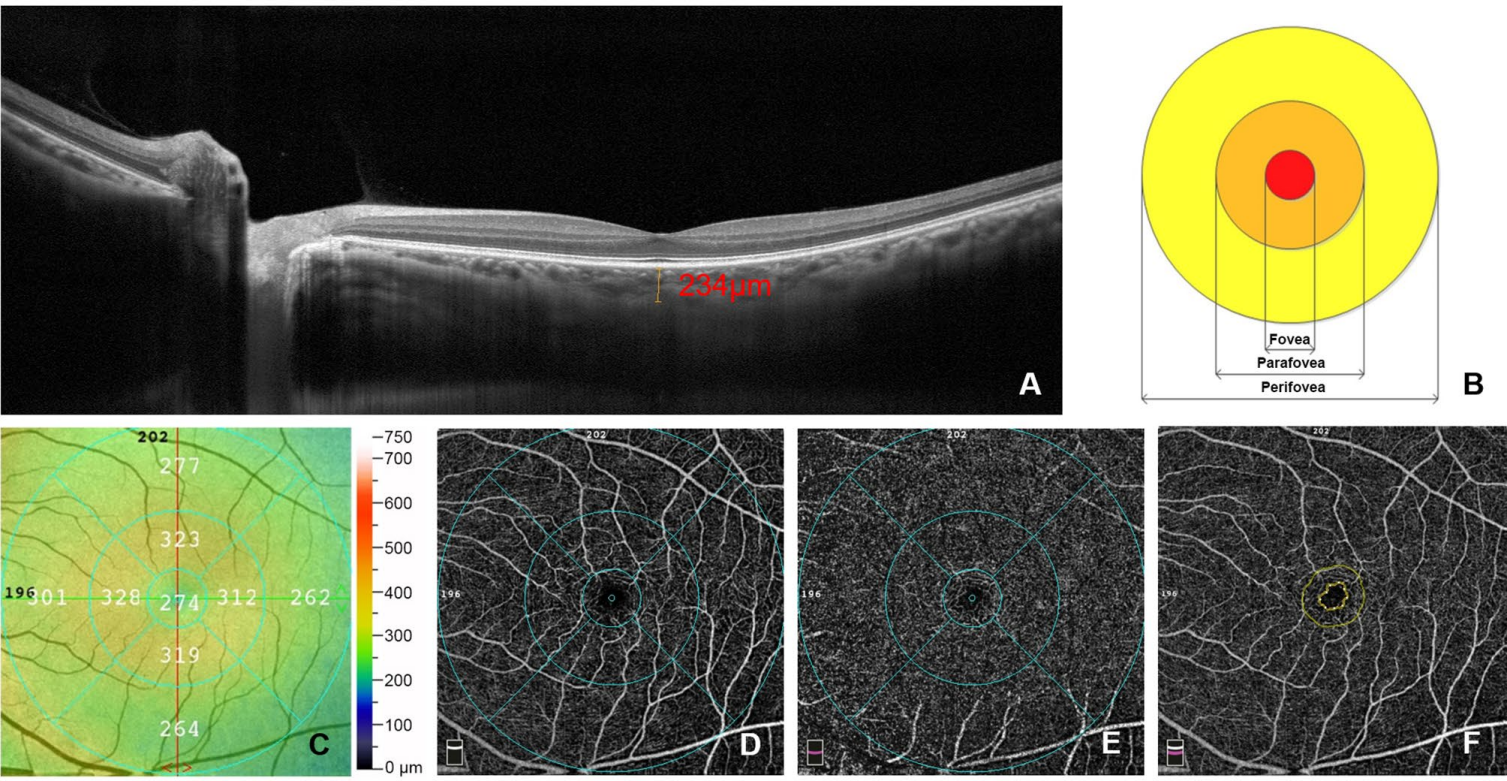
**OCT and OCTA examinations image.** (**a**). SFCT measurement; (**b**). ETDRS grid partition: four subregions centered on the central recess, i.e., the foveal region, the parafoveal region, the perifoveal region, and the entire image; (**c**). RT in the macula;(**d**). SVD in the macula; (**e**). DVD in the macula; (**f**). FAZ

SFCT was defined as the vertical distance between the outer retinal pigment epithelium and the inner scleral border. Each measurement was performed by two experienced technologists, the macular fovea was identified firstly, and then the vertical distance between the outer retinal pigment epithelium and the inner scleral border was measured, these two procedures were repeated for three times and the average value was used for evaluation. A 6 × 6 mm angio OCT scan centred on the fovea was performed on each eye, and the Early Treatment Diabetic Retinopathy Study (ETDRS) grid divided the macula into 3 were as, the foveal region, the parafoveal region, the perifoveal region. The foveal region was a circular were a with a diameter of 1 mm; the diameter of the inner circle of the foveal region was 1 mm, and the outer was 3 mm; the inner diameter of the perifoveal region was 3 mm and the outer diameter was 6 mm. Automated segmentation lines were used to divide the capillary bed into the superficial vessel density (SVD) and deep vessel density (DVD). SVD was automatically segmented from the inner limiting membrane (ILM) to the inner plexiform layer (IPL). DVD was automatically segmented from IPL to the outer plexiform layer (OPL). Using the “density” function of the software can automatically display the percentage of areas occupied by vessels in the macular area. Retinal thickness (RT) was calculated automatically in micrometers. The foveal avascular zone (FAZ) area was calculated automatically in mm^2^ by the software using the “FAZ” measure function. RT, SVD, DVD, and FAZ of the foveal region, the parafoveal region, the perifoveal region, and the entire image were calculated. Data were acquired by the software of the device (see Fig. 1).

### Statistical analysis

Statistical analysis was performed with StataSE 14.0 software (StataCorp, College Station, TX, USA). The Kolmogorov-Smirnov test was used to test the normal distribution of all variables. The continuous variables were summarized using mean ± standard deviation (SD). Measurements of ocular parameters and systemic factors before and after blood donation were compared using the paired-samples t-test when the variables were normally or approximately normally distributed, and the Wilcoxon signed-rank test for correlated samples when the variables did not conform to a normal distribution. Correlations between changes in the indicators were analysed by Pearson correlation analysis when the variables were normally or approximately normally distributed, and by spearman correlation analysis when the variables did not conform to a normal distribution. Multiple stepwise linear regression analysis was used to assess the factors influencing the changes in each index of the choroid and retina.

## Results

### General data

The total number of blood donor volunteers included in the study was 28 people with 56 eyes, including 15 males and 13 females, age ranging from 21 to 48 years. Thirty-two eyes were moderate myopic (-3.00 D to -6.00 D), and the remaining 24 eyes were diagnosed with presbyopia or emmetropia. The axial length was 24.03 ± 1.89 mm (Mean ± SD). The blood donation volume of all the volunteers was 200ml.

### Changes in SBP, DBP, IOP, OPP, and correlation between BP and IOP changes in volunteers before and after blood donation

The paired-samples t-test indicated that IOP got significantly lower 24 h after blood donation (*P* = 0.006, 18.2 ± 3.95 mmHg versus 16.0 ± 4.38 mmHg), while no significant difference existed in the comparisons of SBP, DBP, and OPP at 30 min after blood donation or 24 h after blood donation (*P* > 0.05) (see Table 1).

**Table 1:** Changes in SBP, DBP, IOP, and OPP before and after blood donation

	Before blood donation	30 min after blood donation		24 h after blood donation	
	Mean (SD)	Mean (SD)	*P* ^*^	Mean (SD)	*P* ^*^
**SBP**	126.07 (16.25)	126.21 (16.5)	0.975	121.46 (21.5)	0.369
**DBP**	77.93 (13.25)	75.04 (9.75)	0.357	75.5 (15.25)	0.527
**IOP**	18.2 (3.95)	18 (3.98)	0.790	16 (4.38)	***0.006***
**OPP**	45.07 (6.89)	43.39 (8.51)	0.253	46.73 (10.34)	0.320

**Abbreviations**: DBP: diastolic blood pressure; IOP: intraocular pressure; OPP: ocular perfusion pressure; SBP: systolic blood pressure; SD: standard deviation

^*^ Comparison with corresponding indicators before blood donation

Spearman’s correlation analysis showed that IOP changes at 24 h after blood donation was negatively correlated with SBP (*r* = -0.268, P = 0.046), while no correlation existed between IOP changes and SBP at 30 min after blood donation, or IOP changes and DBP changes (*P* > 0.05) (see Table 2).

**Table 2:** Correlation between BP and IOP changes before and after blood donation

	30 min after blood donation		24 h after blood donation	
	r	*P*	r	*P*
**Change in SBP**	0.156	0.252	-0.268	***0.046*** ^*^
**Change in DBP**	0.256	0.057	-0.164	0.226

**Abbreviations**: DBP: diastolic blood pressure; SBP: systolic blood pressure

^*^P-value at the 0.05 level (two-tailed) with significant correlation

### Changes in SFCT, RT and SVD, DVD, and FAZ before and after blood donation

There was no significant difference in the SFCT and FAZ of volunteers at both 30 min and 24 h after blood donation compared with that before blood donation (*P* > 0.05). For RT, SVD, and DVD at the foveal region, the parafoveal region, the perifoveal region, and the entire image, there were no significant between those before blood donation and 30 min or 24 h after blood donation (*P* > 0.05) (see Table 3).

**Table 3:** Comparison of morphology and blood flow status of the choroid and retina before and after blood donation

	Before blood donation	30 min after blood donation		24 h after blood donation	
	Mean (SD)	Mean (SD)	*P* ^*^	Mean (SD)	*P* ^*^
**SFCT**	243.91 (45.42)	246.43 (44.7)	0.768	248.85 (44.8)	0.564
**RT**					
foveal	242 (39.5)	242.5 (39.5)	0.947	240.5 (39.75)	0.842
parafoveal	328.5 (28.75)	328.1 (32.1)	0.931	327.5 (29.5)	0.856
perifoveal	289 (31)	288.5 (30.25)	0.931	289 (31.75)	1.000
entire image	290 (34.25)	289 (37.25)	0.883	289 (36.75)	0.882
**SVD**					
foveal	17.59 (7.4)	17.61 (6.77)	0.988	18.43 (5.71)	0.503
parafoveal	50.3 (18.75)	49.05 (20.2)	0.735	50.75 (19.45)	0.901
perifoveal	49.2 (13.23)	49.3 (12.33)	0.967	49.5 (13.43)	0.905
entire image	48.35 (14)	48.65 (13.78)	0.909	48.85 (13.58)	0.848
**DVD**					
foveal	33.45 (15.38)	31.05 (19.3)	0.468	33.8 (17.43)	0.911
parafoveal	52.2 (18.9)	51 (19.48)	0.741	51.9 (18.4)	0.932
perifoveal	46.35 (17.78)	47.95 (17.4)	0.631	47.3 (16.48)	0.770
entire image	46.05 (16)	46.65 (17.53)	0.850	46 (15.48)	0.987
**FAZ**	0.324 (0.129)	0.316 (0.127)	0.742	0.317 (0.135)	0.780

**Abbreviations**: DVD: deep vascular density; FAZ: foveal avascular were a; RT: retinal thickness; SD: standard deviation; SFCT: subfoveal choroid thickness; SVD: retinal superficial vascular density

^*^ Comparison with corresponding indicators before blood donation

### Changes in BCVA before and after blood donation

The BCVA before blood donation was 1.01 ± 0.12, no BCVA changes existed in each volunteer at 30 min or 24 h after blood donation, thus there were no changes in BCVA before and after blood donation (*P* > 0.05).

## Discussion

Our study observed and analysed the short-term effects of blood donation on morphology and blood flow of the retina and choroid in healthy volunteers. The results indicated that 200ml blood donation caused IOP reduction at 24 h, which was negatively correlated with SBP, while it did not affect the SBP, DBP, or OPP. Moreover, 200ml blood donation did not influence the morphological and blood flow status of the choroid and retina, as no significant difference existed in the OCT and OCTA indexes like SFCT, RT, SVD, DVD, and FAZ. The visual acuity was not affected either.

### The relationship between BP and IOP

We found that IOP got significantly lower 24 h after blood donation, which was significantly correlated with the reduction of SBP, while no significant difference existed in the comparisons between SBP, DBP, or OPP. These findings verified the theory that the reduction in circulating blood volume might trigger the pressure receptor-mediated sympathetic nervous system immediately, thus heart rate and peripheral vascular resistance would increase to restore the MAP, and perfusion pressure in organs such as the eye would be restored. Although the SBP did not change significantly, it still caused the reduction of IOP at 24 after blood donation, which might be due to the fact that blood pressure changes lead to changes in ciliary blood pressure, then regulates the ultrafiltration capacity of aqueous humor and leads to the changes in IOP[10–13]. While it was really interesting that the IOP reduction at 24 h was negatively correlated with SBP changes, we did review the related studies but no proper explanation was found. We thought the reason might be that, although the SBP showed a trend of reduction at 30 min and 24 h, it might had a transient increase as the blood loss might trigger the transient stress response, this hypothetical transient SBP increase might reduce the reduction of IOP, thus the IOP reduction at 24 h was negatively correlated with SBP changes. This was only a hypothesis, future study which evaluate more time point should be conducted to verify this theory.

### The response of BP and IOP to blood loss

After donating 200ml of blood in healthy individuals, there will be a small decrease in overall blood volume. Class I haemorrhage occurs when the blood loss is 0–15% of the total blood volume, and minimal physiological changes occur at this level. A patient may exhibit mild anxiety, but heart rate, blood pressure and peripheral circulation largely remain unchanged[14]. This is consistent with our research results. A small decrease in blood volume will reduce the area of aortic pressure receptors, and arterial pressure reflex can be activated without detectable changes in arterial pressure[15]; Meanwhile, the tense state of volunteers during blood donation can also cause sympathetic nerve activation[16]. Animal experiments have shown that sustained stimulation (>60 min ) of the ocular sympathetic fibers can evoke a decrease in IOP[17]. Our study shows that there is no significant change in IOP after 30 min of blood donation, while the decrease in IOP after 24 h of blood donation may be related to sustained stimulation of the sympathetic nervous system by low blood volume. Of course, due to our small sample size, more samples need to be included to clarify.

### Effects of changes in BP, IOP, and OPP on the choroid and retina

Although volunteers showed some degree of changes in BP and IOP at 30 min or even 24 h after blood donation, there was no effect on the OPP or visual acuity of the patients. It might be because the relationship between intraocular blood flow and OPP was auto-regulated under normal conditions[3, 18]. Autoregulation was the ability to maintain a relatively constant level of blood flow in the presence of changes in OPP and changes in metabolic demand. Increases in MAP caused by pressure and exercise[19, 20], decrease in nocturnal arterial pressure, and diurnal variations in IOP[21] might cause changes in OPP, followed by local vasoconstriction or dilatation leading to a reciprocal increase or decrease in vascular resistance, thus maintaining a constant nutrient supply and forming the so-called auto-regulatory response. This defect in autoregulation might play an important role in the pathophysiology of ocular vascular diseases[22]. Our study verified that the blood donation of 200ml might not cause a significant influence on the SBP, DBP, or OPP, the changes in IOP at 24 h was also safe with no harmful side effect. We admitted that adding the analysis of heart rate and MAP would make the study more comprehensively, while the heart rate was not measured when we collecting the data, this was the disadvantage of the study and we will evaluate this index in the future research.

### Changes in the choroid before and after blood donation

The choroid was innervated by parasympathetic, sympathetic, and trigeminal sensory nerve fibers. Parasympathetic innervation could dilate blood vessels and increase choroidal blood flow, while sympathetic innervation could constrict blood vessels and decrease choroidal blood flow, and sensory nerves could centrally transmit pain and temperature information and locally act on vasodilation and increased choroidal blood flow. Neuromodulation played an important role in choroidal blood flow regulation[23, 24], and therefore choroidal autoregulation was more pronounced when OPP was elevated or decreased. We, therefore, focused on changes in SFCT, which might indirectly suggest the state of subfoveal choroidal perfusion[9, 25–27]. Our study verified that SFCT was not affected by 200ml blood donation, which might be related to the auto-regulatory function of the choroid. In contrast, Hatice Bilge Araz-Ersan et al.[1] found that SFCT in blood donation volunteers decreased significantly at 1 and 2 h after blood donation and returned to the normal range after 3 h of blood donation. This was inconsistent with our results, probably due to the fact that the blood volume donated by the patients in this study was 200 ml, while it was 500 ml donated in their literature. The reproducibility of the observed index changes needed to be verified in studies with equivalent conditions. Although the OCTA imaging was based on the vessel density, which could not really reflect the blood flow volume directly, it could still illustrate the state of intravascular perfusion in retina and choroid, and reflect the changes of blood volume. Besides, the OCTA device we used was Optovue RX (Optovue Inc. Fremont, Califonia, USA), which mainly provide solid measurement of retinal vessels. While it could not provide measurement of choroid, even the SFCT was manually measured and evaluated. With the update of our OCTA device, we would conduct further related research with more choroidal indexes.

### Changes in the retina before and after blood donation and their influencing factors

The retina was one of the neural tissues with the highest metabolic demand, and its blood flow was supplied by the retinal vascular and choroidal vascular systems. In contrast to the choroidal vessels, retinal vessels lack autonomic innervation except for the central retinal artery, and retinal blood supply is often maintained at a stable level during changes in systemic blood pressure, suggesting that there is also a self-regulatory mechanism for retinal blood perfusion[28]. It had been shown that small retinal arteries maintain stable retinal blood flow by vasoconstriction during increased OPP, and this auto-regulatory function might be due to local factors[29]. It had also been demonstrated that the decrease in OPP due to an increase in IOP causes the small retinal arteries to dilate, which might be due to the regulation of myogenic factors[30]. This was because the increase in IOP leads to a decrease in the transmural pressure of the small retinal arteries, which was necessary to maintain the stability of retinal blood flow during the decrease in OPP[31].

Our study verified the auto-regulatory function in retinal vascular systems, 200ml blood donation did not cause significant changes in RT, SVD, and DVD: For RT, SVD, and DVD at the foveal region, the parafoveal region, the perifoveal region, and the entire image, which also proved the safety of proper blood donation on the ocular system.

### Limitation

However, we admitted that several limitations existed. The best way to explain the inverse correlation between SBP and IOP was to measure SBP over 24 h, which was not measured in the present study. Besides, only statistically significant change might not justify a cause and effect relation and may be by chance findings. Future studies were warranted to focus on this issue.

## Conclusions

In conclusion, our study showed that 200 ml blood donation was noted to be associated with statistically significant IOP reduction at 24 h, while SBP, DBP, or OPP was not affected. The blood flow of the retina and choroid or the visual acuity did not change significantly after the blood donation. Larger studies with different volumes of blood donation were needed to further analysis the effect of blood donation on ocular parameters.

## Data Availability

The datasets used and/or analysed during the current study were available from the corresponding author on reasonable request.

## Abbreviations

AL: Axial length
BCVA: Best corrected visual acuity
CSC: Central serous chorioretinopathy
DBP: Diastolic blood pressure
DVD: Deep vessel density
FAZ: Foveal avascular were a
IOP: Intraocular pressure
MAP: Mean arterial pressure
OCTA: Optical coherence tomography angiography
OCT: Optical coherence tomography
OPP: Ocular perfusion pressure
RT: Retinal thickness
SBP: Systolic blood pressure
SD: Standard deviation
SFCT: Subfoveal choroid thickness
SVD: Superficial vessel density
